# Supplementary material for: High-Throughput Sequencing Reveals Regional Diversification of Cucurbit-Infecting Begomoviruses in Eastern Saudi Arabia
Source: Viruses. 2026 Jan 5;18(1):75. doi: 10.3390/v18010075 (PMC12846466; doi:10.3390/v18010075)
Supplement: Supplementary file 1 [file viruses-18-00075-s001.zip › viruses-4044968-supplementary.pdf]

High-throughput Sequencing Reveals Regional Diversification of Cucurbit-Infecting Begomoviruses in Eastern Saudi Arabia

Muhammad N. Sattar[1,*], Sallah A. Al Hashedi[1], Mostafa I. Almaghasla[2,3], Sherif M. El-Ganainy[2,3], Adil A. Al-Shoaibi[1,4] and Muhammad Munir[5,*]

[1] Central Laboratories, King Faisal University, P.O. Box 420, Al-Ahsa 31982, Saudi Arabia

[2] Department of Arid Land Agriculture, College of Agricultural & Food Sciences, King Faisal University, P.O. Box 420, Al-Ahsa 31982, Saudi Arabia

[3] Pests and Plant Diseases Unit, College of Agriculture and Food Sciences, King Faisal University, P.O. Box 420, Al-Ahsa 31982, Saudi Arabia

[4] Department of Physics, College of Science, King Faisal University, P.O. Box 400, 31982 Al Ahsa, Saudi Arabia

[5] Date Palm Research Center of Excellence, King Faisal University, P.O. Box 420, Al-Ahsa 31982, Saudi Arabia

* Correspondence:

mnsattar@kfu.edu.sa (M.N.S); mmunir@kfu.edu.sa (M.M)

Tel: +966582123443 (M.N.S); +966540229813 (M.M.)

Supplementary Table S1: Field sampling, PCR screening, and NGS identification of cucurbit begomoviruses from Al-Ahsa and Qatif, Saudi Arabia

No.	City	Location	Coordinates	Sample	Sample ID	Host	PCR	Coat Protein	NGS
**1**	Al-Ahsa	DPRC	25.460° N, 49.564° E	8	SqH1	Zucchini	+	ToLCOMV	TYLCV
**2**				9	SqH5	Zucchini	+		
**3**		Almoussa	25.248° N, 49.608° E	**11**	SqH41	Zucchini	+	ToLCOMV	WmCSV OL416207 OL416208
**4**				**12**	SqH37	Zucchini	+	ToLCOMV	
**5**				18	SqH32	Snake gourd	-		
**6**				19	SqH33	Snake gourd	+	ToLCOMV	TYLCV OL416209
**7**		Qatar Road	25.178° N, 49.438° E	**25**	SqH10	Zucchini	+	ToLCOMV	
**8**				**26**	SqSK2	Zucchini	+	ToLCOMV	
**9**				**27**	SqSK4	Zucchini	-		
**10**				**28**	SqSK7	Zucchini	+	ToLCOMV	TYLCV
**11**				**29**	SqSK10	Zucchini	+	ToLCOMV	
**12**		Al-Uyun	25.579° N, 49.567° E	**36**	Sq3SK15	Zucchini	+	ToLCOMV	
**13**				37	Sq4SK16	Zucchini	+	ToLCOMV	
**14**	Qatif	Aljaysh	26.556° N, 49.996° E	4	SqA8my	Zucchini	+	WmCSV	WmCSV DNA-A DNA-B
**15**				6	SqA9my	Zucchini	+		
**16**		Al-Zaayir	26.556° N, 49.996° E	16	SqAl32	Snake gourd	-		
**17**		Almghaslah	26.556° N, 49.996° E	30	SqA2	Snake gourd	+	ToLCOMV	WmCSV DNA-A, DNA-B ToLCPalV DNA-A, DNA-B
**18**				51	SqS4	Snake gourd	-	ToLCOMV	
**19**		Sanabil	26.556° N, 49.996° E	38	SqS40	Snake gourd	-		
